# Reply to: “Thermal artefacts in two-photon solar cell experiments”

**DOI:** 10.1038/s41467-019-08704-1

**Published:** 2019-02-27

**Authors:** Shigeo Asahi, Takashi Kita

**Affiliations:** Department of Electrical and Electronic Engineering, Graduate School of Engineering, Kobe University, 1-1 Rokkodai, Nada, Kobe, 657-8501 Japan

**Replying to** C. C. Phillips *Nature Communications* 10.1038/s41467-018-07166-1 (2019)

Two-step photon up-conversion solar cells (TPU-SCs) are among the most promising photovoltaic devices^1,2^. In our previous works, we demonstrated the increase in the open-circuit voltage as well as the short-circuit current induced by the additional infrared (IR) light^1^. Phillips suggested that this phenomenon could be attributed to the sample heating^2^. Here, we present additional data suggesting that photoexcitation might be the cause. In the TPU-SC, the up-conversion is achieved by a series of two-step photoexcitation. By absorbing a below-gap photon, an electron transits from the valence band to the conduction band of the narrow bandgap semiconductor. Upon absorption of another below-gap photon, the electron accumulated at the hetero-interface is further pumped into the conduction band of the wide bandgap semiconductor. This ideal TPU process, following the absorption of two below-gap photons, produces additional photocurrent and boosts the photovoltage depending on the band offset at the hetero-interface. In this Reply to Matters Arising, we discuss the excitation power dependence of the external quantum efficiency (EQE) observed in TPU-SC and the efficient intraband excitation at the hetero-interface.

We measured the change in the EQE (ΔEQE) of TPU-SCs with InAs quantum dots (QDs) as a function of interband excitation power density. Here, ΔEQE was defined as the difference between the EQE obtained with and without the 1300 nm laser diode (LD) illumination. We conducted a systematic experiment to study the excitation power density dependence of the increased short-circuit current, Δ*J*_SC_, and the ΔEQE. Detailed measurements taken at 300 K are presented in Fig. 1. We used a 784-nm continuous wave (CW) LD and a 1300-nm CW LD for the interband and the intraband excitations, respectively. With increasing interband excitation density, the Δ*J*_SC_ increases sub-linearly and tends to deviate from the power law at high-excitation density above ~10 mW cm^−2^. Thereby, ΔEQE significantly decreases with the interband excitation density. This set-up reproduces the results reported in our original article. Note that, in this experiment, we did not use a simulator and, indisputably, there were no artefacts of the simulator’s sensitive reference channel optical detectors with scattered laser light. Furthermore, we did not perform a signal modulation technique for the photocurrent detection, because the lifetime of electrons separated from the holes can be extended to milliseconds^3^.

**Fig. 1:**
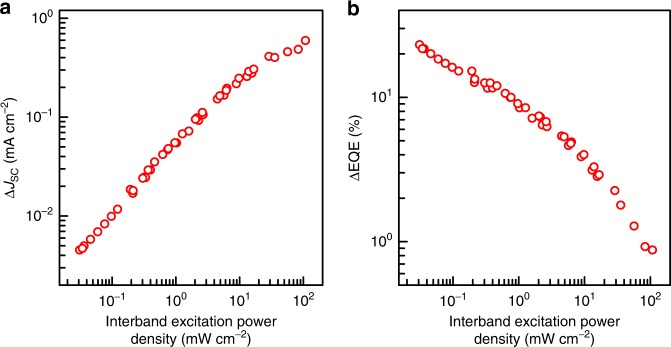
Interband excitation power density dependence of two-step photon up-conversion current at 300 K. **a** Change in the short-circuit current density (Δ*J*_SC_). **b** Change in the external quantum efficiency (ΔEQE); the wavelength and excitation power density of the second intraband excitation light were 1300 nm and 300 mW cm^−2^, respectively

Here, we discuss the effects of thermal artefacts on the results. If Δ*J*_SC_ is produced by the sample warming under the 1300-nm LD illumination, Δ*J*_SC_ is given by

1 $${\mathrm{\Delta }}J_{{\mathrm{SC}}} = J_{{\mathrm{SC}}}\left[ {\exp \left( { - \frac{{E_{\mathrm{a}}}}{{k_{\mathrm{b}}T + k_{\mathrm{b}}{\mathrm{\Delta }}T}} + \frac{{E_{\mathrm{a}}}}{{k_{\mathrm{b}}T}}} \right) - 1} \right],$$

where *k*_b_ is the Boltzmann constant, *E*_a_ is the activation energy, *T* is the temperature, and Δ*T* is proportional to the 1300-nm LD power density. This gives an almost linear relationship in the measured region, as shown in Fig. 7b of the original article. However, the measured intraband excitation power density dependence in Fig. 7b was sublinear. Therefore, Δ*J*_SC_ is not necessarily caused by thermal artefacts. We considered the possibility that the sublinear dependence of Fig. 7b arises from a change in the electron density at the hetero-interface. Figure 7d of the original article illustrates the dependence of the power-law index *n* on the reverse-bias voltage in the relationship of the intraband excitation power density and Δ*J*. As the reverse-bias voltage increases, *n* increases and approaches unity. The space charge reduced by the intraband excitation weakens the electric field at the hetero-interface, resulting in a sub-linear response to the excitation density caused by the weaker electric field reducing the carrier collection efficiency of the TPU. We have recently reported the bias voltage dependence of *n* in detail (for example, ref. ^4^). The result is shown in Fig. 2, which reproduces the data of Fig. 7d of the original article. It is interesting to note that *n* quickly returns to unity by injecting carriers at the forward bias above ~0.4 V. When enough carriers are injected, the influence of the accumulated electrons on the electric field near the hetero-interface is weakened. Thus, the sub-linear relationship between Δ*J* and the interband excitation power density in Fig. 1 answers all questions. Accumulated electrons at the hetero-interface significantly influence the local electric field near the hetero-interface, which changes the carrier collection efficiency of the TPU. Therefore, Δ*J* is determined by the number of intraband excited carriers and their collection efficiency. Detailed analysis and the physics behind Fig. 1 will be discussed in our upcoming paper.

**Fig. 2:**
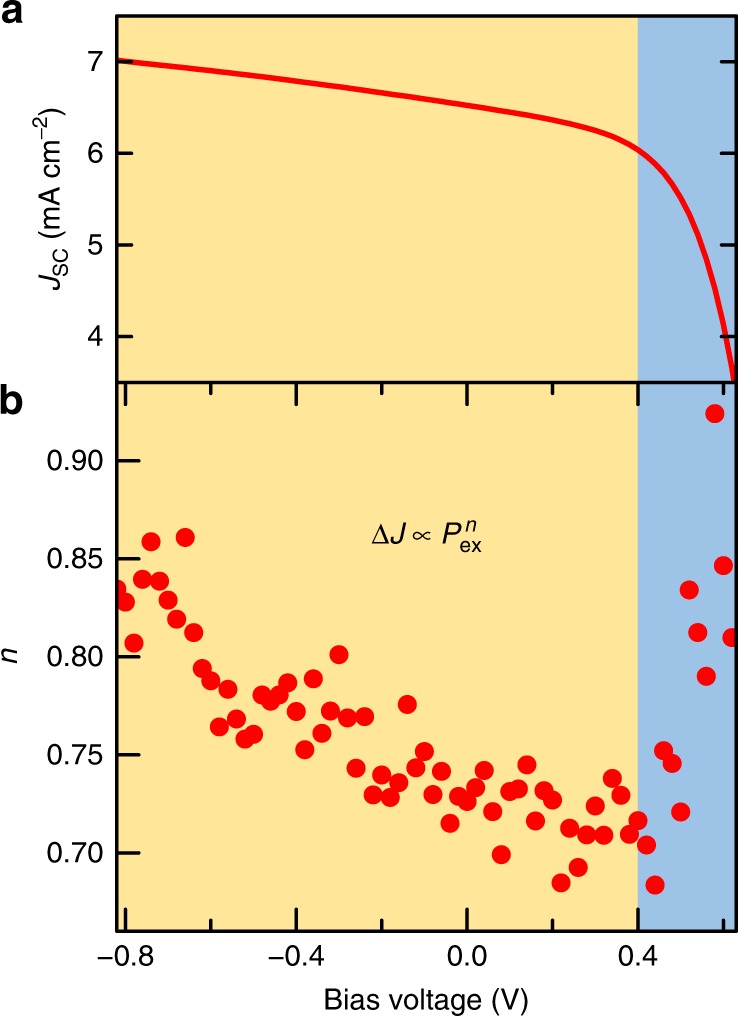
Bias voltage dependence of two-step photon up-conversion properties at 297 K. **a** Short-circuit current density (*J*_SC_). **b** *n* value of ${\mathrm{\Delta }}J_{{\mathrm{SC}}} \propto P_{{\mathrm{ex}}}^n$, where Δ*J*_SC_ is the change in the short-circuit current density, and *P*_ex_ is the 1300 nm excitation power density. The wavelength and excitation power density of the first interband excitation light were 780 nm and 110 mW cm^−2^, respectively. *P*_ex_ was varied in the range 1–320 mW cm^−2^

We next focus on the efficient intraband excitation occurring at the hetero-interface. Free carrier absorption should be considered at least in the TPU-SC without InAs QDs. If only the free carrier absorption occurs in the ‘bulk’ portion, the accumulated electron density becomes considerably high and causes a lower bound. It is well known that the wave-function of the accumulated electrons can generally penetrate the barrier layer. The wave-function penetration depth depends on the electric field at the hetero-interface. The penetrated component contributes to the transition from the confined level to the conduction band edge of AlGaAs, which improves the excitation cross-section and, therefore, lowers the estimated electron sheet density. Such a mechanism can contribute to the improvement of the intraband excitation at the hetero-interface. Further experiments and theoretical work are necessary to unveil the efficient photoexcitation at the hetero-interface, which is the most important and interesting mechanism in TPU-SC.

The intermediate-band SC includes intermediate states in the bandgap. By absorbing a below-gap photon, an electron transits from the valence band to the intermediate band. Upon absorption of another below-gap photon, the electron is further excited into the conduction band. This ideal TPU process, following the absorption of two below-gap photons, produces additional photocurrent without degrading the photovoltage. However, it is well known that thermal coupling between the conduction band and the intermediate band gives rise to a reduction of *V*_OC_. Many studies discussing the *V*_OC_ reduction have been published^5^. Similar processes occur at the hetero-interface of the TPU-SC. The photo-carrier generation of the TPU-SC without IR illumination indicates that the conduction bands of AlGaAs and GaAs are thermally coupled. The thermal coupling becomes stronger with rising temperature, which causes the change in *V*_OC_ according to Eq. (4) of the original article. This analytical model suggests that Δ*V*_OC_ depends on the interband carrier density. We recently confirmed the strong dependence of the change in *V*_OC_ on the interband excitation density. The different trends, compared in Fig. 7e of the original article, are essential to demonstrate the contribution of the optical excitation process.

Finally, we would like to comment on the data error of the current–voltage (*J*–*V*) curve shown in Fig. 7a of the original article. The *J*–*V* curve was drawn by smoothly connecting current density measured at discrete voltages at a step of every 0.02 V (see Fig. 3), and there is no real data point in the area magnified in the inset of the original Figure 7a. To better demonstrate the precision of *J*–*V* curve and thus *V*_OC_ that can be extracted from our original measurements, we replot the magnification of *J*–*V* curve around a zero current density in the inset of Fig. 3 here by highlighting the uncertainty induced by the errors in the original data. The shaded areas around the *J*–*V* curve represent the standard error of the current density. It is clear that the uncertainty in our *V*_OC_ measurements is substantially smaller than the difference between *V*_OC_s that can be extracted from the raw data at three irradiation conditions.

**Fig. 3:**
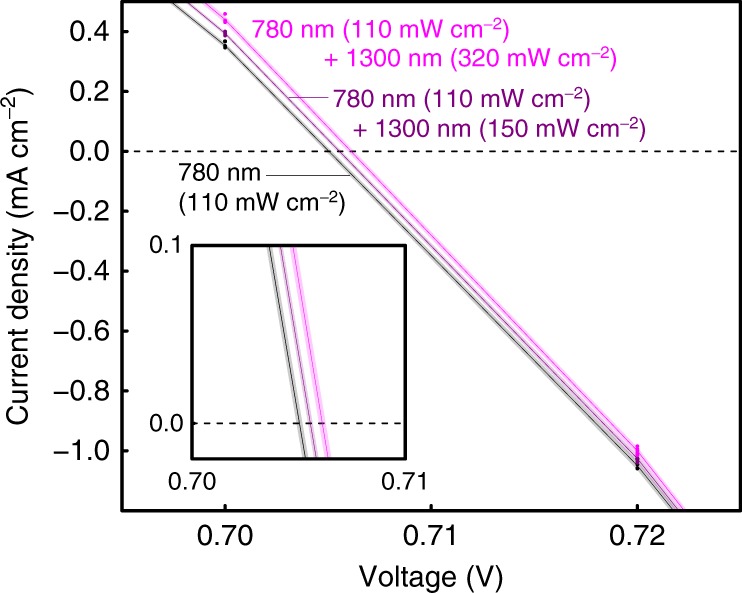
Current–voltage curve under the two-colour illumination of 780 nm and 1300 nm lasers: the solid circles indicate data points at 0.70 V and 0.72 V. The shaded areas represent the width of the standard error evaluated using the data. The inset shows the magnification of the open-circuit voltage curve as indicated in the inset of Fig. 7a of the original article. All data are the same as the data in the Fig. 7a, b, and c of the original article

## Data Availability

The data that support the findings of this study are available from the corresponding author upon request.
